# Health interventions for migrants and refugees in host Southeast Asian countries: a systematic review: Khatia Antia

**DOI:** 10.1093/eurpub/ckac131.510

**Published:** 2022-10-25

**Authors:** J Sappyabanphot, TT Aye, P Shreedhar, Z Wasko, K Antia, V Winkler

**Affiliations:** Heidelberg Institute of Global Health, Heidelberg University Hospital, Heidelberg, Germany

## Abstract

**Background:**

Understanding the different types of health interventions that have been conducted for migrants and refugees is crucial for the improvement and implementation of future health interventions for these populations. This systematic review aimed to identify and to look at the scope and outcomes of health interventions focused on migrants and refugees in the main host counties in Southeast Asia which are Thailand, Singapore, and Malaysia.

**Methods:**

This study was conducted in line with the PRISMA guidelines and its protocol has been submitted to PROSPERO. The following databases were searched until June 2021: PubMed, Web of Science, Science Direct, Cochrane, and Google Scholar. Studies were excluded if: 1) they were conducted outside Thailand, Singapore, and Malaysia; 2) had only had qualitative results; 3) were non-peer reviewed; 4) not written in English.

**Results:**

The search yielded 8,266 studies, out of which 33 were included in the review. The majority of the studies (79%) were conducted in Thailand of which most were focused on migrants or refugees from Myanmar (85%). Besides two randomized controlled trials (RCTs) of mental health interventions, most Thai studies were observational (81%) and focused on infectious disease-related interventions (33%) or the evaluation of health-related programs (29%). Six studies were conducted in Malaysia (18%) of which 4 assessed mental health interventions in refugees. Three of these studies were RCTs, whereas 1 was an observational study. Only 1 study was situated in Singapore and was an RCT evaluating treatments for COVID-19 in migrant workers. Even in studies with similar interventions, outcomes were too diverse to conduct a meta-analysis.

**Conclusions:**

The low number of studies highlights the gap in literature on health interventions for migrants and refugees, especially in Malaysia and Singapore. More rigorous and cohesive intervention-related research needs to be conducted in Southeast Asia.

**Key messages:**

• More intervention-related research for migrant and refugee populations in the main Southeast Asian host countries is needed.

• Interventions for migrant and refugee populations in host countries in Southeast Asia often do not follow the gold standard RCT study design, limiting the knowledge on their effectiveness.

